# Postoperative analgesic effect of dezocine combined with sufentanil: A propensity score matching study

**DOI:** 10.1097/MD.0000000000043041

**Published:** 2025-06-27

**Authors:** Xinguo Li, Caiyan Yang, Hui Yan, Xiaobo Suo, Zong Ma, Daqing Xu

**Affiliations:** a Department of Pharmacy, Ningxia Hui Autonomous Region Ning’an Hospital, Yinchuan, Ningxia, China; b Department of Pharmacy, Yinchuan First People’s Hospital, Yinchuan, Ningxia, China.

**Keywords:** analgesics, dezocine, propensity score matching, sufentanil

## Abstract

Postoperative pain can produce a variety of adverse reactions, prolong postoperative recovery time and length of hospital stay and increase medical costs. Postoperative analgesia methods are different; dezocine combined with sufentanil is one such method, but its the efficacy remains uncertain. Therefore, we investigated the clinical efficacy and medical costs of dezocine combined with sufentanil for postoperative analgesia. A retrospective observation and analysis were conducted on all patients admitted to a tertiary hospital in Ningxia Hui Autonomous Region from March 1, 2022 to March 3, 2024 for surgery and postoperative analgesia. Patients were divided into 2 groups according to the treatment of postoperative pain: treatment group (dezocine combined with sufentanil) and control group (conventional sufentanil analgesia). Propensity score matching was performed for sex, age, weight, and analgesic pump parameters (total drug dose, first drug dose, duration of analgesia, patient-controlled drug dose, and infusion rate). After propensity score matching, 1178 patients were included. There were no significant differences in the baseline characteristics between the 2 groups, and the histogram and jitter plots of the propensity score distribution indicated good matching. There was no significant difference in clinical analgesic efficiency (88.5% vs 86.8%), 24 hours analgesic score (0.95 vs 0.95), 48 hours analgesic score (0.58 vs 0.58) and 72 hours analgesic score (0.37 vs 0.37) between the 2 groups. The overall incidence of adverse events (11.4% vs 14.9%, *P* = .07) was similar between the 2 groups. However, the subgroup analysis showed that the incidence of nausea and vomiting in the control group was higher than that in the treatment group (8.5% vs 5.6%, *P* = .02), and the incidence of somnolence in the control group was lower than that in the treatment group (2.0% vs 4.4%, *P* = .02). There was no significant difference in the incidence of vertigo or other adverse reactions between the groups. In addition, the cost of analgesics in the treatment group was significantly higher than that in the control group (the cost of analgesics: 484.74 ± 177.39 in the treatment group VS 83.35 ± 14.55 in the control group), and the difference between the 2 groups was statistically significant (*P* < .001). Dezocine combined with sufentanil can be used for postoperative analgesia, but this analgesic method has no obvious advantage over sufentanil alone, In addition, it increases the healthcare burden on patients.

## 1. Introduction

Postoperative pain is injurious pain caused by stimulation of the body, which generally does not last for more than 7 days. Pain is the response of the body to trauma or disease. In medical behavior, surgery is the main cause of pain in patients. Statistically, most patients experience significant postoperative pain.^[1,2]^ Postoperative pain can cause accelerated heart rate, vasoconstriction, increased cardiac load, increased myocardial oxygen consumption, gastrointestinal dysfunction, urinary retention, and serious adverse events such as myocardial ischemia, heart failure, and respiratory depression.^[3]^ At the same time, it will also affect the early postoperative activities and rehabilitation of patients, such as pain not being effectively controlled at the beginning of the operation. Continuous pain stimulation can cause pathological remodeling of the central nervous system, and acute pain may develop into chronic pain that is difficult to control.^[4]^ Therefore, for the control of postoperative pain, in addition to the high requirements of doctors’ medical skills, the correct choice of postoperative analgesia and good use of postoperative analgesic drugs are also crucial. It can not only avoid excessive postoperative pain and reduce the occurrence of adverse reactions, but also has great significance for patients in shortening the length of hospital stay, reducing medical costs, and reducing postoperative rehabilitation.^[5]^ Dezocine is a mixed-acting opioid receptor agonist that acts on the central nervous system. It has different effects on κ, μ, and δ receptors and is increasingly used for postoperative pain management.^[6–8]^ As a μ-receptor agonist, sufentanil has a good analgesic effect, and its combination with μ-receptors will produces side effects, such as intestinal motility inhibition, nausea and vomiting, and respiratory depression.^[9]^ Studies have shown that dezocine combined with sufentanil can reduce postoperative pain, adverse reactions and other advantages, but the sample size of the study is relatively small, mostly about 30 cases. This study aimed to evaluate the real-world performance of dezocine combined with sufentanil. It is expected that the findings of the study will provide evidence-based evidence for the clinical use of medication and save healthcare expenditure for patients.

## 2. Methods

### 2.1. General characteristics

The study followed the reporting guidelines of Strengthening the Reporting of Observational Studies in Epidemiology. This study was approved by the Ethics Committee of First People’s Hospital of Yinchuan (KY-2024-002). The basic information and pain pump parameters of patients who underwent surgery in our hospital between March 2022 and March 2024 and required postoperative analgesia were retrospectively collected. Inclusion criteria: patients with complete postoperative PCIA records, American Society of Anesthesiologists grade i to iii, age range 16 to 65 years old. The exclusion criteria were as follows: ≥20% missing data, severe liver and kidney dysfunction, long-term chronic pain (such as autoimmune diseases and rheumatoid arthritis), or neuropathic pain.

### 2.2. Grouping of patients

The treatment group was treated with dezocine (National Medicine Permit No. H20080329) combined with sufentanil (National Medicine Permit No. H20054172), while the control group was treated with sufentanil alone.

### 2.3. Data collection and processing

Clinical data were retrospectively collected, exported from the hospital information system, and manually entered. By combining the purpose of this study and the variables involved in the medical records, we extracted the following information. We collected clinical data retrospectively and exported it from the hospital information system and entered it manually. The persons collecting the receipts were professionally trained, while the data were always collected by the same 2 persons under the supervision of their supervisors. Demographic characteristics: sex, age and body weight; analgesic pump parameters: total dose, first dose, duration of analgesia, patient-controlled dose, infusion rate, diagnostic information, type and dosage of drugs used in the analgesic pump, ADR and its occurrence time. VAS was used to record the time and score. Mean imputation was used for missing data of <20%. The data in this study were obtained from a real-world clinical. In the data processing process, sensitive data fields were de-identified and anonymized, and the relevant regulations of information security technical specifications and medical big data security management were strictly followed. All of these data can be obtained by contacting the corresponding author.

### 2.4. Evaluation of postoperative analgesic effect

The postoperative VAS was used to evaluate the analgesic effect of the drugs, with higher scores indicating more severe pain. The analgesic score and adverse drug reactions, such as nausea, vomiting, abdominal distension, and respiratory depression, were recorded within 72 hours after surgery.

Clinical effective rate: the VAS score was 0 at 72 hours after the operation, and no other drugs or solvents were added or subtracted during the use of an analgesic pump. In addition, the VAS score was recorded 24, 48 and 72 hours after the operation, and the analgesic score was recorded 24, 48 and 72 hours after the operation to evaluate the analgesic effect. The incidence of ADR during postoperative analgesia was recorded, the safety of the clinical use of drugs was evaluated, and the economy was evaluated by least cost analysis:


$$\begin{array}{l} \begin{array}{ll} & Clinical\;ef\;f\;icacy\;(\%)=number\;of\;ef\;fective\;cases\;\\ & /total\;number\;of\;cases\times 100\%.\; \end{array} \end{array}$$

$$\begin{array}{l} \begin{array}{ll} & The\;number\;of\;adversereactions(n)\;\\ & =the\;sum\;of\;all\;adverse\;reactions.\; \end{array} \end{array}$$

$$\begin{array}{l} \begin{array}{ll} & Incidence\;of\;adverse\;reactions\;(\%)\;\\ & =number\;of\;patients\;with\;adverse\;reactions\;\\ & /total\;number\;of\;patients\times 100\%.\; \end{array} \end{array}$$

### 2.5. Statistical analysis

SPSS22.0 (version 22.0.0; IBM Corporation, Chicago) software was used to complete the statistical work of SPSS data, and the count data were expressed as frequencies and percentages. The chi-square test, corrected chi-square test, or Fisher’s test were used for comparison between groups. The measurement data were first tested for normality, and those in line with a normal distribution were statistically described as mean ± standard deviation (*x* ± *s*). Two independent sample *t*-test were used for comparison between groups, a paired sample *t*-test were used for comparison between groups, and median (quartile) [M (P25, P75)] was used to describe those not in line with normal distribution. A nonparametric test was used for analysis. Hypothesis tests (standardized differences) were used to test the homogeneity of the data before and after correction, and to evaluate the balance of covariates between groups. Statistical significance was set at *P* < .05. Propensity score matching (PSM) was used to reduce the influence of selection bias and potential confounding factors. Using 1:1 nearest neighbor matching, a histogram and jitter plot of the propensity score distribution were drawn to evaluate the matching effect. After PSM, 589 patients in each group were included in the study.

## 3. Results

### 3.1. Comparison of general characteristics

Finally, 1738 patients were included in the study, including 1082 patients in the dezocine combined with sufentanil group and 656 patients in the sufentanil group.

**Figure 1.**
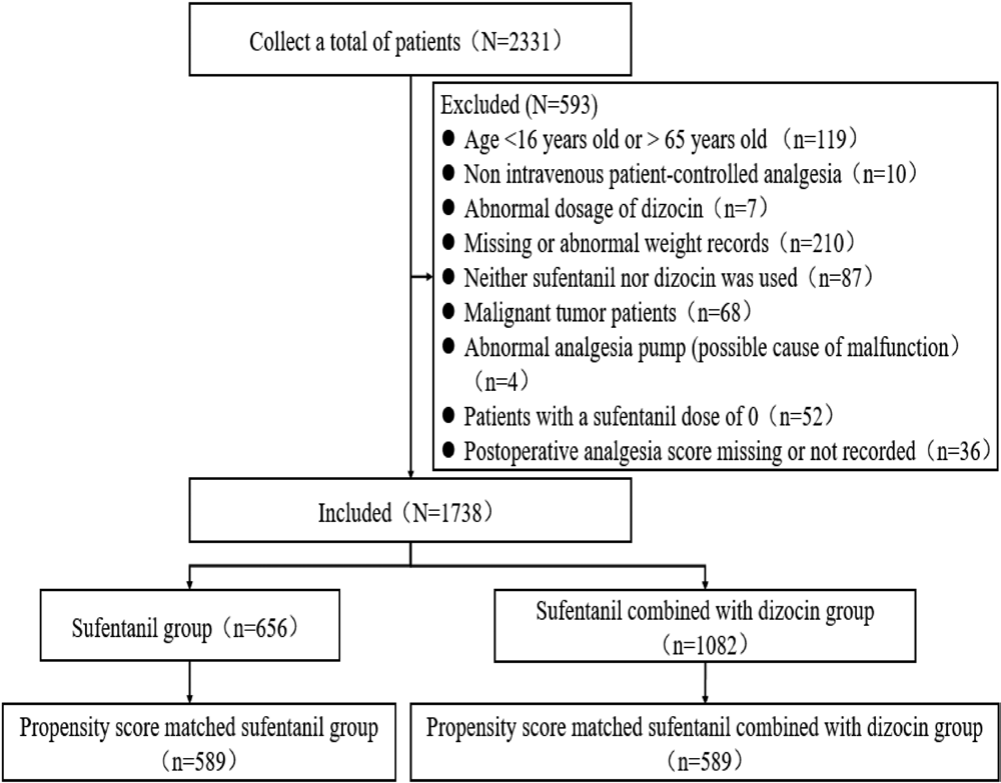
Flow chart of the study process.

Figure 1 shows the study flow chart. The baseline clinical characteristics of the 2 groups were not balanced. To exclude the influence of confounding factors on the outcome, PSM was used to balance and compare the clinical baseline data of the 2 groups. There were no significant differences between the 2 groups in terms of sex, age, weight, type of anesthesia, time of analgesia, total drug dosage, first dose, patient-controlled drug dosage, infusion speed, and lockout time (*P* > .05; Table 1). Finally, 1178 cases were successfully matched, including 589 in the treatment group and 589 in the control group.

**Table 1:** Comparison of clinical baseline after propensity score matching in 2 groups of patients with postoperative intravenous self-controlled analgesia.

Baseline characteristics	After propensity score matching (1178)			
	Control group (589)	Treatment group (589)	Statistical value	*P* value
Sex (%)			0.115	.734
Male	146 (24.8)	141 (23.9)		
Female	443 (75.2)	448 (76.1)		
Age	35 (29, 52)	36 (28, 53)	−0.488	.626
Weight	69 (60, 77)	69 (60, 77)	−0.672	.502
Anesthesia (%)			0.239	.625
General anesthesia	201 (34.1)	209 (35.5)		
Semianesthesia	388 (65.9)	380 (64.5)		
Analgesic time	72 (68, 72)	72 (68, 72)	−1.057	.290
Doses (mL)	150 (150, 200)	150 (150, 220)	−0.221	.825
First dose	4 (3, 5)	4 (3, 5)	−1.784	.074
Self-control dose	1.5 (1, 2)	1.5 (1, 2)	−0.473	.636
Self-control dose	2 (2, 3)	2 (2, 3)	−1.121	.903
Lock time	15 (15, 20)	15 (15, 20)	−0.386	.700

**Figure 2.**
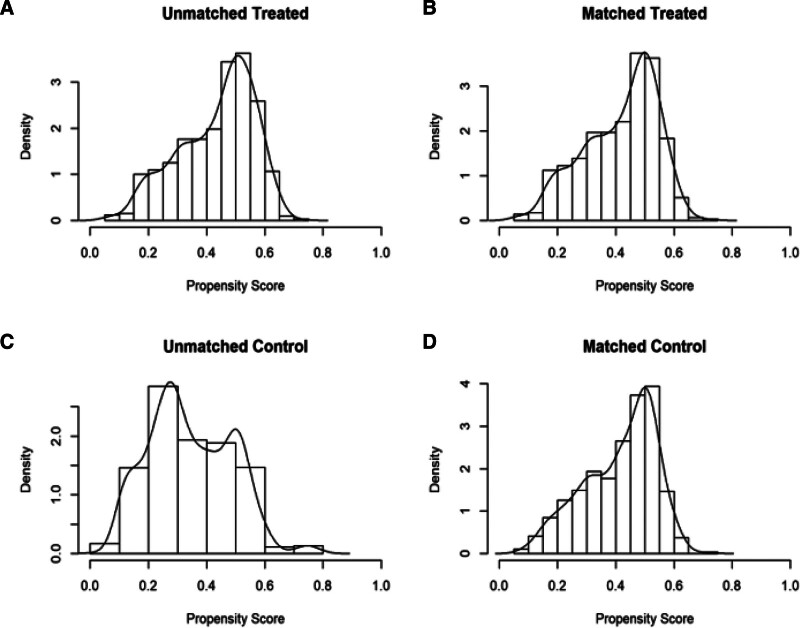
Histograms of effects before and after matching (A) shows the data effect bar chart of the treatment group before matching, (B) shows the data effect bar chart of the treatment group after matching, (C) shows the data effect bar chart of the control group before matching, and (D) shows the data effect bar chart of the control group after matching).

Figure 2 shows a histogram of the propensity score distribution, which is used to show the similarity of the data score distribution before and after matching. If the area shapes of the upper and lower images tend to be the same after matching, it indicates that the matching effect is good.

**Figure 3.**
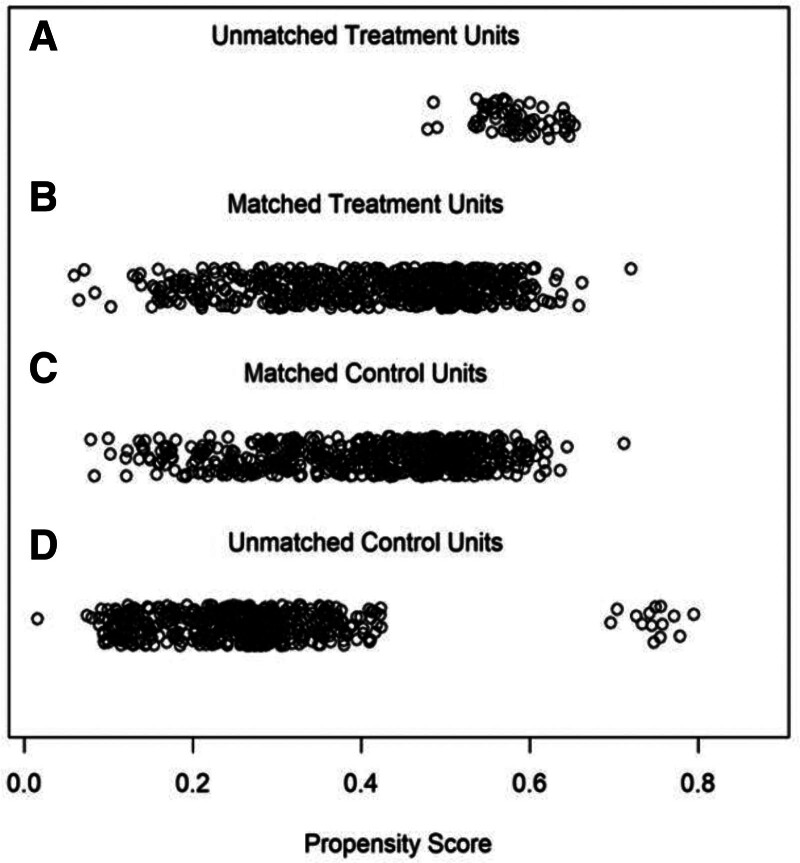
The jiggle dot plot before and after propensity score matching matching (A) shows the data jitter plot removed during the matching process of the treatment group, (B) shows the data jitter plot after matching the treatment group, (C) shows the data jitter plot removed during the matching process of the control group, and (D) shows the data jitter plot after matching the control group).

Figure 3 shows a jitter graph of the propensity score distribution. This study used a 1:1 ratio, and the matching tolerance was 0.2 the area of each point in the score distribution graph represents the weight, which shows that the baseline difference areas of the 2 groups were balanced and comparable after matching, and the matching effect was good.

Table 2 shows the effective rate of postoperative analgesia and the cost of analgesia after 3 days in the 2 groups before and after PSM. Before PSM matching, the effective rates of analgesia in the treatment group and control group were 87.5% and 88.0%, respectively, 3 days after surgery, and the difference was not statistically significant (*P* = .789). After matching, the effective analgesia rates in the treatment group and the control group were 86.8% and 88.5%, respectively, and the difference was not statistically significant (*P* = .377). The cost of analgesia in the 2 groups was statistically different before and after PSM (*P* < .001).

**Table 2:** Comparison of clinical efficiency and analgesic cost before and after propensity score matching between the 2 groups.

	Groups	Effective	Ineffective	Effective rate	Cost
Before propensity score matching	Control group (656)	577	79	88.0%	83.14 ± 14.33
	Treatment group (1082)	947	135	87.5%	504.43 ± 162.99
	Statistical value			0.071	
	*P* value			.789	<.001
After propensity score matching	Control group (589)	521	68	88.5%	83.35 ± 14.55
	Treatment group (589)	511	78	86.8%	484.74 ± 177.39
	Statistical value			0.782	
	*P* value			.377	<.001

Table 3 presents the VAS pain scores within 24, 48, and 72 hours after surgery in the 2 groups. After PSM adjustment, there was no significant difference in the VAS scores between the 2 groups at any time point (The *p*-values are respectively *P* = .506, *P* = .504, *P* = .136).

**Table 3:** Comparison of visual analogue scale analgesia scores between the 2 groups within 72 hours after surgery.

	Groups	24 h	48 h	72 h
Before propensity score matching	Control group (656)	0.95 (0, 1)	0.58 (0, 1)	0.37 (0, 0.37)
	Treatment group (1082)	0.95 (0, 1)	0.58 (0, 1)	0.37 (0, 0.37)
	Statistical value	−0.492	−0.013	−0.654
	*P* value	.623	.990	.513
After propensity score matching	Control group (589)	0.95 (0, 1)	0.58 (0, 1)	0.37 (0, 0.37)
	Treatment group (589)	0.95 (0, 1)	0.58 (0, 1)	0.37 (0, 0.37)
	Statistical value	−0.665	−0.667	−1.490
	*P* value	.506	.504	.136

Table 4 shows the incidence of adverse reactions in the 2 groups. There was no significant difference in the overall incidence of adverse reactions between the 2 groups (*P* = .07).

**Table 4:** Incidence of total adverse reactions before and after propensity score matching in the 2 groups.

	Groups	No adverse drug reaction	Adverse drug reaction	Adverse drug reaction rate (%)	*P* value
Before propensity score matching	Control group (656)	577	79	12.0	.007
	Treatment group (1082)	900	182	16.8	
After propensity score matching	Control group (589)	522	67	11.4	.070
	Treatment group (589)	501	88	14.9	

Table 5 shows the results of the subgroup analysis of adverse reactions. After PSM, the incidence of nausea and vomiting in the control group was higher than that in the treatment group (8.5% vs 5.6%, *P* = .029), and the incidence of somnolence in the control group was lower than that in the treatment group (2.0% vs 4.4%, *P* = .021). There was no significant difference in the incidence of vertigo or other adverse reactions between the groups (*P* = .817).

**Table 5:** Subgroup analysis results of adverse reactions before and after propensity score matching in the 2 groups.

Adverse reactions		Groups	No adverse drug reaction	Adverse drug reaction	Adverse drug reaction rate (%)	*P* value
Nausea and vomiting	Before propensity score matching	Control group (656)	596	60	9.1	.148
		Treatment group (1082)	1004	78	7.2	
	After propensity score matching	Control group (589)	539	50	8.5	.029
		Treatment group (589)	558	31	5.6	
Vertigo	Before propensity score matching	Control group (656)	624	32	4.9	.350
		Treatment group (1082)	1011	71	6.5	
	After propensity score matching	Control group (589)	563	26	4.4	.497
		Treatment group (589)	558	31	5.3	
Lethargy	Before propensity score matching	Control group (656)	644	12	1.8	<.001
		Treatment group (1082)	1026	56	5.2	
	After propensity score matching	Control group (589)	577	12	2.0	.021
		Treatment group (589)	563	26	4.4	
Others	Before propensity score matching	Control group (656)	647	9	1.4	.111
		Treatment group (1082)	1055	27	2.5	
	After propensity score matching	Control group (589)	580	9	1.5	.817
		Treatment group (589)	579	10	1.7	

## 4. Discussion

Our study found that dezocine combined with sufentanil for postoperative analgesia has no obvious advantage over sufentanil alone, and increases the medical burden of patients. Postoperative pain is difficult to effectively control with a single drug, but whether it should be combined with dezocine for analgesia and the dose of dezocine combination still needs to be considered. Previous small sample and retrospective studies have shown that dezocine can reduce sufentanil-induced cough and significantly improve patient satisfaction; however, the relevant small sample studies are not consistent with real-world applications. We believe that this study provides a strong supplement to previous research. Opioids are traditional postoperative analgesic drugs, with morphine being the most common, followed by fentanyl. In recent years, sufentanil and remifentanil have been widely used.^[10]^ Sufentanil, a derivative of fentanyl, is a potent opioid and a highly selective μ-receptor agonist with analgesic efficacy 1315 times that of morphine and 5 to 10 times that of fentanyl.^[11]^ As a widely used postoperative analgesic drug,^[12]^ compared with tramadol, dezocine, ketorolac aminotriol and other common postoperative analgesics, sufentanil has the characteristics of good analgesic effect, rapid onset, wide safety range, long duration of action, and no accumulation in the body. At the same time, sufentanil lozenges have been developed for postoperative analgesia, which has been proven to be safe and effective, and can also reduce the economic burden of patients.^[13]^ In addition, the hemodynamic effects of sufentanil in children are also relatively positive.^[14]^ However, adverse reactions, such as nausea, vomiting, and drowsiness, can also occur. In the past, dezocine was generally considered to selectively activate κ receptors and antagonize μ receptors, but recent studies have shown that dezocine actually partially antagonize κ receptors and partially activates μ receptors, so that nausea and vomiting adverse reactions in the gastrointestinal tract can be reduced accordingly. However, dezocine antagonize receptors while activating μ receptors, resulting in excessive sedation, drowsiness, and other adverse reactions.

## 5. Limitations

This study had certain limitations. First, the PSM matching method was used to control the baseline balance of the 2 groups, but there were still some potential factors that were not found. Second, the dose of dezocine was fixed in this study, and the optimal dose of dezocine combined with sufentanil remains unclear. In future studies, we plan to adjust the ratio of the 2 drugs to achieve the maximum clinical effect. Finally, this was a retrospective, single-center, real-world study, and although the sample size was relatively large, there were potentially missing data. We are preparing to conduct a prospective, multicenter, real-world study for further investigation. Although our study has some limitations, it can still provide medication guidance and possible information for clinical physicians.

## 6. Conclusions

The effect of dezocine combined with sufentanil is equivalent to that of sufentanil alone in postoperative analgesia. In addition, compared with sufentanil alone, dezocine combined with sufentanil does not show obvious advantages in inhibiting postoperative adverse reactions, while increasing the medical costs of patients.

## Acknowledgments

We would like to thank Dr Chunsheng Li for his invaluable help with statistical analysis and editing.

## Author contributions

**Data curation:** Hui Yan, Xiaobo Suo, Zong Ma.

**Supervision:** Caiyan Yang, Daqing Xu.

**Writing – original draft:** Xinguo Li.
